# Transdiagnostic prevention in youth mental health, Part II: interventions

**DOI:** 10.1038/s41386-025-02234-9

**Published:** 2025-10-07

**Authors:** Nicole R. DeTore, Oyenike Balogun, Karmel W. Choi, Daphne J. Holt

**Affiliations:** 1 Department of Psychiatry, Massachusetts General Hospital, Boston, MA USA; 2 Harvard Medical School, Boston, MA USA; 3 Department of Natural and Applied Sciences, Bentley University, Boston, MA USA

**Keywords:** Risk factors, Outcomes research

## Abstract

The emerging consensus regarding transdiagnostic and dimensional models of psychopathology has important implications for the development of effective approaches for early intervention and prevention of psychiatric conditions. Consistent with biological, epidemiological and clinical evidence, transdiagnostic prevention focuses on reducing risk factors and/or preventing outcomes that are shared across populations who may be at risk for a range of psychiatric disorders. This narrative review describes some of the efforts made to date to develop and test interventions aiming to support universal, selective, and indicated prevention of psychiatric conditions in a transdiagnostic manner, during childhood, adolescence, and early adulthood. An evidence-based transdiagnostic approach to the prevention of mental illness in youth, that accounts for the existing body of knowledge about transdiagnostic risk factors as well as constraints impacting “real world” feasibility of these approaches, has the broad potential to ultimately reduce the incidence and severity of psychiatric illnesses.

## Introduction: transdiagnostic prevention in psychiatry

The need for prevention-focused mental health programs for youth is becoming more urgent, as a global youth mental health crisis [1] and the related workforce shortage in mental health care [2, 3] continue to worsen. A widespread implementation of successful preventive interventions that decreases the incidence or severity of mental illnesses would have far-reaching benefits in terms of reducing suffering, disability and the economic consequences of these often lifelong conditions, which typically begin during the first few decades of life.

Much of the prior research on primary prevention in mental health has focused on preventing the onset of specific Diagnostic and Statistical Manual of mental disorders (DSM)-defined disorders or related outcomes. For instance, there have been many randomized control trials (RCTs) examining the efficacy of interventions for preventing major depression [4], anxiety disorders [5], eating disorders [6], postpartum depression [7, 8], substance use disorders [9], and psychotic disorders [10]. However, a prevention approach in psychiatry that is, to a varying extent, *transdiagnostic* (aiming to prevent more than one disorder) has also gained traction in recent years [11], particularly as evidence for the potential cost-effectiveness of this strategy has accumulated [12–14].

Historically, at first, transdiagnostic *treatments* of existing psychiatric disorders were developed, emerging from a recognition of the extensive comorbidity and symptom overlap of psychiatric conditions, particularly across internalizing (e.g., mood and anxiety) disorders, leading to the development of transdiagnostic psychotherapy approaches for treating these conditions [15]. More recently, *prevention* frameworks in psychiatry have also become increasingly transdiagnostic, with the ultimate goal of reducing the onset of multiple, potentially mechanistically-related, psychiatric illnesses in individuals with transdiagnostic risk factors [16, 17]. Empirically, the evidence for shared genetic and environmental risk factors and overlapping neurobiological and psychological manifestations of psychiatric illnesses (see companion paper to this article, Part 1 [18]) may ultimately lead to further refinement of transdiagnostic preventive approaches, with the development of interventions that target such shared risk factors. In this narrative review, we describe approaches to transdiagnostic prevention in psychiatry that have been developed and tested to date, highlighting key examples with discussion of their strengths and limitations, and suggestions for future directions for the field.

A wide range of therapeutic approaches have been employed in transdiagnostic prevention programs, including behavioral interventions, psychotropic medications, neuromodulation, neurofeedback, and supplements [19–21]. Given that behavioral approaches have undergone the largest amount of testing and typically have favorable risk-benefit ratios – an important feature of a prevention program for populations with low to moderate levels of risk—we have limited the focus of this article to *behavioral* interventions used for transdiagnostic prevention of mental illnesses in youth.

Preventive interventions, including transdiagnostic ones, can be provided to an entire population regardless of risk level (*universal prevention*), to individuals with one or more relatively “silent” risk factors such as a family history of a disorder, or a history of early adversity (*selective prevention*), or to those with subclinical symptoms of illness (*indicated*) [22]. Each of these three types of approaches can play a role in a tiered or stage-based approach to the prevention of mental illnesses [17, 23]. Below, we describe examples of each of these strategies as applied to a transdiagnostically at-risk population and/or to prevent transdiagnostic outcomes. Given evidence that the majority of psychiatric illnesses begin early, during the first two decades of life [24], prevention-focused interventions in psychiatry are often designed for pre-adolescent children (and/or their caregivers), adolescents, or young adults. Because the field of transdiagnostic prevention in psychiatry is still at a relatively early stage, we have mainly described published RCTs, plus a few illustrative, single-arm studies of emerging, novel approaches.

## Universal prevention

### Overview

Universal prevention programs, which, by definition, target heterogeneous, transdiagnostically at-risk general population samples, including individuals with minimal levels of risk, are typically aimed at improving the psychological well-being of an entire group of individuals within a particular community setting, regardless of risk level. These types of programs include social-emotional or anti-bullying curricula in schools, wellness programs in workplaces, public health awareness campaigns providing information about the early signs of addiction, depression, anxiety, or suicidality, and screenings in pediatric and primary care practices for maltreatment or depression [25]. Such programs have the potential to impact population levels of psychiatric disorders if implemented widely, akin to the impact on general health of taxes on soda and alcohol, public information campaigns about the benefits of good nutrition and exercise or the health risks associated with smoking, and hypertension and cancer screening. The potentially powerful effect of such universally-delivered interventions can be attributed in part to the counter-intuitive “prevention paradox”—that the majority of cases of a disease arise from individuals at moderate or low risk, with only a minority of cases arising from individuals at high risk [26]. Thus, an intervention that reduces a highly prevalent risk factor, even one associated with a small degree of risk, can have a large impact on disease incidence. However, the effectiveness of universal interventions may be limited by the large samples typically needed to observe benefits [16]. Conversely, a universal preventive intervention that reduces the risk for and, ultimately, the incidence of, a number of disorders (i.e., with a transdiagnostic impact) could overcome this limitation [11], particularly since, in aggregate, mental illnesses occur in a large portion ( > 25%) of the population [27, 28].

### School-based universal prevention

Universal prevention programs have often been deployed and tested in K-12 schools and colleges, since these are convenient and feasible settings to deliver mental health-focused material to a large number of young people. Also the inclusion of all youth in a particular setting, such as a class or afterschool program, has the benefit of avoiding the potentially stigmatizing effect of selecting youth with certain risk factors or symptom profiles for a mental health intervention. Many of these programs have focused on reducing or preventing common symptoms (rather than disorders), such as anxiety [29], depression [4], suicidality [30], and stress [31].

In addition, some of these programs have focused on a particular behavior that is indirectly linked to mental health, and this may be a particularly effective strategy in youth. For example, one meta-analysis examining the effects of 69 school-based anti-bullying interventions [32] found that they significantly decreased rates of bullying and levels of anxiety and depression. Such “indirect” approaches [33], that focus on addressing specific behaviors or problems that are of concern for youth (e.g., insomnia, overall stress, test anxiety, procrastination, conflicts with peers and other relationship issues, social media use), which are often accompanied by symptoms of depression or anxiety, may be appealing to adolescents and young adults, particularly in light of the minimal stigma associated with many of these topics.

In any school-based programs, teachers are trained to deliver the intervention [34]. One well-studied program that has used this approach is a universal prevention program called the Good Behavior Game (GBG), which has been mainly deployed in young children [35, 36]. GBG focuses on classroom management techniques that employ rules and rewards, aiming to promote positive student behavior [37]. The research on this program has shown that GBG is effective in reducing, as well as preventing (during middle school and early adulthood), aggressive behavior and related outcomes [38, 39]. For example, one study which followed 1,197 first and second grade students who had been randomized to either GBG, a reading curriculum, or standard programming [39], found that, compared to the students assigned to the other conditions, those who had received GBG had lower rates of aggressive behavior, substance use, and anti-social behavior 14 years later.

Mindfulness-based universal preventive interventions have also been studied extensively within schools. One systematic review and meta-analysis of 61 studies of group-based mindfulness interventions conducted across the world (including a total of 6207 students) found evidence for small but significant beneficial effects of these programs on cognitive and socio-emotional outcomes [40]. However, one of the largest RCTs of a mindfulness intervention delivered by teachers (with 8376 adolescents; mean age: 12 years) failed to show mental health benefits of the intervention. This study (the MYRIAD trial) tested an intervention with 10 structured sessions, comprised of psychoeducation, discussion, and brief mindfulness exercises, that were delivered during one semester of school, and compared this intervention to a usual social emotional learning curriculum, with randomization occuring at the school level [41]. While this program showed promise in terms of the feasibility of implementing a structured mindfulness-based intervention within schools, there was no evidence for beneficial effects of the intervention on symptoms of depression, social-emotional behavioral functioning, and wellbeing at the one-year follow-up time point [42]. The trial’s investigators offered several possible explanations for these negative results, such as that the dose of mindfulness training may have been too small; the usual social emotion learning curriculum (the control condition) was beneficial; some students benefited from the active intervention while others did not need it or even experienced worsening when trained to attend to their thoughts and feelings, as observed in other trials [43]; and the meta-cognitive capacities needed to successfully use mindfulness techniques may not be fully developed during early adolescence [42]. Perhaps most importantly, the low acceptability ratings and low engagement levels with the home practice component of the mindfulness intervention highlight the importance of collaboration (co-design) with the targeted youth population when developing preventive behavioral interventions.

Another example of a school-based universal prevention program is the Climate Schools Combined (CSC) intervention, a combination of a substance use and a mental health intervention. CSC aims to concurrently prevent substance abuse, anxiety, and depression via 18 sessions (each comprised of 20 minutes of an individual, online cartoon-based activity, plus 20 minutes of a teacher-led interactive classroom activity) focused on psychoeducation and cognitive behavioral skills [44, 45]. When tested in a cluster-randomized school-based trial that enrolled 6386 adolescents (mean age: 13.5 years), a significantly reduced likelihood of drinking and of experiencing increased anxiety and depression, relative to the mental health-focused control condition, was observed up to 2.5 years after the trial [44]. However, no differences between groups was observed 5 and 6 years later, suggesting that these benefits were temporary [45]. Also, the mental health component alone showed no benefits when compared to a usual health education class [46].

### Schools versus other settings

School-based interventions offer many appealing features and potential solutions to the current gaps in mental health care for youth, such as scalability and access to the children who may benefit. Given that children typically spend most of their time in school, it is unsurprising that youth obtain mental health services within schools at a rate equal to or higher than in clinical settings [47]. However, schools often lack the staffing and other resources needed to deliver mental health-related interventions [48]. Also, there is some evidence that school-based programs delivered by non-school staff with some mental health care training (e.g., clinicians, researchers, or graduate students) are more effective, in terms of leading to reductions in anxiety and depression, than those delivered by teachers or other school staff [49, 50]. These findings may be related to some reluctance of students to share their mental health-related experiences with their own teachers.

Delivering universal mental health-promoting interventions in a range of community settings, such as libraries, youth centers, primary care clinics, sports programs, or online, in parallel with school-based programs, may be an effective approach for providing preventive interventions in settings where youth spend their unscheduled (non-school) time. The value of this pathway is supported by the success of community-based youth programs established outside of schools that focus on both promoting mental health and addressing other needs of youth (see below).

### Future directions

Strengths and limitations of universal prevention approaches in mental health, including questions about their overall effectiveness, have been extensively discussed and debated recently [51–54]. One commonly cited limitation is that the training, oversight, and resources dedicated to these programs can be highly variable. Another challenge is that some of the youth receiving these interventions may not need them or show low engagement [55], leading to a variable, often limited impact. These issues may account, in part, for why several prior reviews have described mixed results for these programs, i.e., that they generally have small effects [49, 56] and often lack rigor [49, 57–60]. Also, some studies have found that, while some of these interventions may show efficacy in reducing or preventing symptoms of depression [58, 61], there is little evidence that they prevent more serious and chronic mental illnesses, such as bipolar disorder or psychosis [58]. Moreover, one meta-analysis found evidence that universal prevention programs are less effective than more targeted prevention programs [62]. Taken together, studies conducted to date suggest that additional, rigorously controlled studies, accompanied by comprehensive training of facilitators and fidelity monitoring, are needed to determine whether some universal prevention interventions are beneficial and cost-effective. One potential strategy that could be tested further in future studies is to make universal programs voluntary [63], which could increase the likelihood that youth with the most interest or need for these programs receive them. Given the nonstigmatizing, accessible features of these programs, and promising findings for some universal interventions in low and middle income countries [50, 54, 64], efforts to develop highly “youth-friendly” universal prevention programs, perhaps with a greater focus on “indirect” behavioral goals rather than explicit mental health ones, are likely worthwhile. Ultimately, universal and targeted prevention programs (see below) may each contribute in complementary ways to a comprehensive, tiered mental health care program for youth [58, 65].

## Selective prevention

### Overview

Selective prevention strategies focus on individuals with “silent” (asymptomatic) risk factors for illness, such as genetic/familial risk or environmental (e.g., childhood adversity) risk factors, which tend to be transdiagnostic, i.e., they are not specifically linked to elevated risk for developing one psychiatric illness [18]. Selective prevention strategies have not been used frequently in psychiatry, given that these types of risk factors are often both relatively common and only weakly predictive of later onset of an illness. However, it is important to consider that the level of risk for developing *any* mental illness associated with these risk factors is typically quite elevated [18]. For example, one meta-analysis reported that approximately half of people with a parent with schizophrenia, bipolar disorder, or depression will develop one of those illnesses by early adulthood [66]. In addition, individuals with familial risk tend to develop more severe symptoms and have an earlier age of onset than average [67, 68]. Similarly, people with a history of childhood maltreatment are approximately twice as likely to develop any mental illness than those without such a history [69]. Thus, several studies have examined the effects of preventive interventions in young people with a relative with a mental illness or substance use disorder, or in those who have experienced abuse, socioeconomic hardships, or traumatic events [21, 70].

### Family-focused selective prevention

Many selective prevention interventions have focused on very young children and the family unit, and are often conducted either with the parent/guardian alone or with parent/child dyads. These interventions often focus on a range of goals, including psychoeducation, family therapy, communication skills and coping strategies. One example of this type of program is the Attachment and Biobehavioral Catch-up (ABC) intervention [71]. ABC has been tested in parents of children at risk for maltreatment, with most studies recruiting samples referred to child welfare (due to the potential need to remove the child from the home). It is a 10-week program aiming to prevent later psychopathology in infants up to 2 years old, delivered to the parents of the child. A systematic review of ten ABC RCTs found that ABC was effective in improving emotion regulation, decreasing externalizing and internalizing problems, and even preserving child development timelines in the children [72].

Another selective preventive intervention that has been extensively studied is Parent-Child Interaction Therapy (PCIT), a program for children, age 2-7 years, who have experienced abuse [73]. PCIT focuses on both increasing the levels of attachment between parents/guardians and their children, as well as improving the behavior of the child. Through numerous RCTs, PCIT has shown efficacy not only in reducing externalizing behaviors and preventing developmental delays in the at-risk children, but in preventing childhood maltreatment [74].

In addition, a few selective prevention interventions have been developed for youth with familial risk for psychiatric disorders. The Building Regulation in Dual Generations (BRIDGE) program is delivered to mothers experiencing depression who are currently caring for their 2–5 year-old child [38]. This program teaches these mothers dialectical behavioral skills to improve their mental health, with the ultimate goal of also protecting the mental health of their child [39]. There are strong feasibility and acceptability data for this program, but no published RCTs to date [75].

Other studies focused on children with parents diagnosed with a specific psychiatric condition have typically focused on preventing the occurrence of the same disorder in the child (despite the evidence that this type of familial risk is associated with an increased likelihood of developing a range of psychiatric disorders). For example, several studies have demonstrated the efficacy of group-based CBT delivered to adolescents with a parent with a history of depression in preventing the onset of depression in the adolescents [76–78].

Another family-based, selective preventive intervention called Alternatives for Families was designed for children 5-15 years old who have experienced physical abuse. This program focuses on preventing Post-Traumatic Stress Disorder (PTSD) symptoms and disruptive behaviors in these at-risk children, using CBT techniques. Two RCTs conducted with this population found that Alternatives for Families was effective in improving outcomes in both the child (in terms of functioning, anger, depression, aggression and PTSD symptoms) and caregiver (in levels of anger and abusive behavior) up to six months later [79, 80].

Another selective preventive intervention, designed for adolescents, is the World Health Organization’s Early Adolescent Skills for Emotions (EASE) [81]. This intervention is delivered to either the parent, child, or both, and includes seven group-based sessions for the adolescent (10-14 years old), plus two sessions for the parents. When delivered to Burundian refugee families who had been displaced following a violent conflict (and thus at elevated risk for developing psychopathology) [82], EASE was found to improve family relationships and adolescent well-being [83].

Lastly, the Strong African American Families (SAAF) program was designed for African American adolescents and their family members living in communities in the rural American South with high poverty levels [84–86]. Youth living in these communities are at elevated risk for later substance abuse and a range of poor health outcomes. The SAAF intervention consists of 7 weekly meetings in community settings focused on improving parenting, strengthening family relationships and youth competencies. One RCT of SAAF, which enrolled 667 African American mothers and their 11-year old children, found that improvements in parenting mediated beneficial effects of SAAF on the children, compared to an assessment-only control group, on a range of protective factors, including attitudes about early alcohol use and sexual activity, a goal-directed future orientation, and acceptance of parental influence, at the 7-month follow-up assessment [84]. In addition, approximately 5 years following the study, alcohol use had increased at a slower rate in the youth who had participated in SAAF compared to the control group [87]. At 8 years following the study, lower levels of peripheral inflammatory markers were observed in a subset of the SAAF participants compared to control group participants, which was partially attributable to improvements in parenting [88]. Thus, such family-based interventions focused on pre-adolescent youth may have long-term, wide-ranging health benefits. However, additional well-controlled trials, which measure psychiatric and physical health outcomes directly, are needed.

### Future directions

Selective prevention strategies have been generally under-utilized, despite the elevated transdiagnostic risk for developing mental illnesses associated with a wide range of familial and environmental risk factors. This approach should receive greater investment in the future, particularly since selective prevention approaches may have the advantage, in terms of gaining acceptance by the public, of being easily comparable to prevention strategies used in other fields of medicine, such as those deployed in asymptomatic people who have familial risk for serious medical conditions such as cancer, heart disease and stroke. Public education and the development of a standardized screening approach for these silent mental illness risk factors, combined with safeguards that prevent stigmatizing effects of a “positive” screening result, could lead to wider implementation of this strategy. However, similar to universal preventive interventions, the strength of the effects of these programs may be limited when the risk factor is only weakly predictive of the outcome and the outcome has a low incidence rate. Also, the neuro-psychological mechanisms underlying the vulnerability to mental illness may vary within an at-risk group; consequently, the intervention may benefit only a portion of those at-risk. In light of these limitations, selective preventive behavioral interventions may be more likely to succeed if: 1) the intervention targets multiple psychological mechanisms; 2) the risk factor or combination of risk factors are strongly predictive of a poor outcome; and 3) the outcome is transdiagnostic (increasing the strength of this predictive association).

## Indicated prevention

### Overview

Indicated prevention strategies in mental health, which focus on individuals who have early, subthreshold symptoms of psychopathology, have been studied and tested extensively. Most of these programs focus on youth with subclinical symptoms of a specific disorder or a category of related disorders. However, although many of the initial studies of preventive interventions with this design were focused on preventing a specific disorder, such as major depression or schizophrenia, many of these interventions likely had broad, transdiagnostic effects. Regardless, our review (below) of indicated prevention interventions is primarily limited to those programs that were explicitly designed to target a transdiagnostically-defined at-risk population and/or outcome(s).

In order to target individuals with transdiagnostically-defined *risk factors* for mental illnesses [89], studies of indicated preventive interventions have often used a certain threshold score on a general mental health screening instrument, that typically measures a range of symptoms of different psychiatric disorders, or a similar threshold score on a validated personality measure, as the primary eligibility criterion. Participants with scores at or above this threshold, who do not exhibit the severity of symptoms or functional impairment associated with a full syndromal psychiatric illness, are then considered to be at risk for developing an illness.

Transdiagnostically-defined *outcomes* in these studies are typically: 1) levels of two or more symptom types (e.g., anxiety and depression) and/or 2) behavioral outcomes (e.g., school attendance, overall functioning). These outcomes are assessed via interview, self-report and/or parent report. Notably, very few studies of transdiagnostic, indicated preventive interventions identified the onset of a psychiatric diagnosis as the primary outcome. This is likely due to limitations in resources, since the length of longitudinal follow-up and the sample size needed to observe an effect on a categorical outcome such as illness onset requires a substantial investment of effort that occurs infrequently in mental health prevention research.

It is also important to note that most prevention-oriented behavioral interventions represent minor adaptations of interventions used for the treatment of psychiatric disorders, and this is particularly true for indicated preventive interventions (since they focus on mildly symptomatic individuals), including those that are transdiagnostic. For example, the most extensively-studied transdiagnostic behavioral treatment (used most commonly to treat mood and anxiety disorders) is the Unified Protocol (UP) for Transdiagnostic Treatment of Emotional Disorders (https://unifiedprotocol.com), which is currently available in 11 languages and in individual, group, and digital formats [15, 90]. The UP focuses on emotional awareness, flexible thinking, emotion avoidance, emotion-related physical sensations, and emotion-focused exposure [91] and is undergoing testing as a preventive intervention (with mixed, albeit preliminary, results thus far [92, 93]).

### School-based indicated prevention

Similar to universal prevention programs, many indicated prevention interventions have been deployed in primary and secondary schools (K-12), as well as in colleges and universities. One intervention focused on the transdiagnostic prevention of anxiety and mood disorders is the EMOTION program, a CBT-based program for 8–12-year-old children with subsyndromal symptoms of anxiety or depression [94]. In a school-based RCT (with randomization occurring at the school level) conducted with 795 symptomatic children, the children in the EMOTION group showed a significant reduction in their symptoms compared to those enrolled in the control condition (treatment as usual, defined as psychoeducation training of school staff).

As mentioned above, indicated prevention programs often rely on a brief mental health screening process to identify at-risk children experiencing subclinical symptoms of psychopathology. One commonly used screening instrument is the Strengths and Difficulties Questionnaire (SDQ), which assesses emotions, conduct, inattentiveness, peer relationships and prosocial behaviors [95]. One study that used the SDQ to identify at-risk children tested a CBT-based, manualized indicated preventive intervention called Mind My Mind (MMM). This intervention was compared to “management as usual” (MAU, consisting of a choice of counseling, advice, educational support, or no treatment) in an RCT that enrolled 396 at-risk children (ages 6-12, mean: 10 years). Using the SDQ, these children were identified as having symptoms of anxiety, depression or behavioral problems that were still below the threshold needed for a referral to mental health care. Compared to the MAU control condition, the children in the MMM group showed significant improvements in symptoms and functioning as measured by the SDQ (reported by the child), as well as parent-reported outcomes for the child, including depression, anxiety, social functioning, and school attendance, up to 26 weeks later [96].

Another school-based intervention, called Mastermind, has been studied with a single arm design, using the SDQ to identify 80 children (mean age: 12 years) with mild symptoms of psychopathology. These children were enrolled in eight after-school sessions focused on empowerment, self-esteem building and attention bias modification to reduce maladaptive negative thinking. Improvements in self-esteem, anxiety, depressive, and behavioral symptoms occurred following the intervention [97].

A well-studied indicated preventive intervention designed for older adolescents, focused on preventing symptoms of anxiety and depression, is the Structured Material for Therapy (SMART) program, which is a 6-week, 5-module, CBT-based intervention. SMART was studied in a waitlist- controlled RCT of 163 adolescents (mean age: 15.5 years) identified using the SDQ as having mild “emotional problems”. This study found that SMART led to improvements in internalizing symptoms and global functioning at the six months follow-up time point [98].

Personality dimensions have also been used to identify youth who might benefit from an indicated preventive intervention. For example, Preventure is a group-based intervention for adolescents that aims to prevent substance use and psychiatric disorders. An RCT of Preventure used personality dimensions derived from the self-report Substance Use Risk Profile screening battery [99] to identify and enroll 701 students (mean age: 13.4 years) with elevated scores on impulsivity, sensation seeking, negative thinking, and/or anxiety sensitivity. Worsening of psychiatric symptoms was slower in the group that received Preventure up to three years later, compared to a control group which received usual health education [100].

Similarly, an intervention was developed for young adults who were deemed at risk for psychiatric disorders based on a personality assessment. The intervention, called the Personality and Living of University Students (PLUS), is a CBT-based program focused on emotion regulation, self-esteem building, and combating perfectionism, among other goals. In an RCT, 1,047 college students were recruited online based on their scores on a personality instrument (assessing neuroticism, concern with mistakes, doubts about actions, and hopelessness) and were randomized to either receive PLUS or a healthy lifestyle control intervention. Compared to the control, significant improvements were found for those who received PLUS in self-esteem and symptoms of depression and anxiety 12 weeks later [101].

### Family-focused indicated prevention

Indicated prevention approaches for youth with subclinical symptoms of psychopathology have also included family-based interventions. For example, Emotion Focused Skills Training (EFST) is a manualized intervention delivered to parents that aims to promote parental self-efficacy and emotion regulation skills in parents and their children. It does this by helping parents manage their own and their children’s emotions, repair relationship ruptures, set boundaries for their child and foster communication within the family, using psychoeducational material plus explicit facilitation of emotion activation, using evocative experiential techniques. When the full EFST program (psychoeducation and emotion activation) was compared to the psychoeducational component alone, with parents of 236 adolescents experiencing internalizing and externalizing symptoms, the full EFST program was not found to be significantly more effective than the psychoeducational component in reducing the children’s symptoms and preventing them from reaching a clinical threshold of symptom severity one year later [102]. However, both the full EFST and psychoeducational component alone were associated with reductions in internalizing and externalizing symptoms in the adolescents at the one year follow-up time point.

### Resilience-focused indicated prevention

**Fig. 1:**
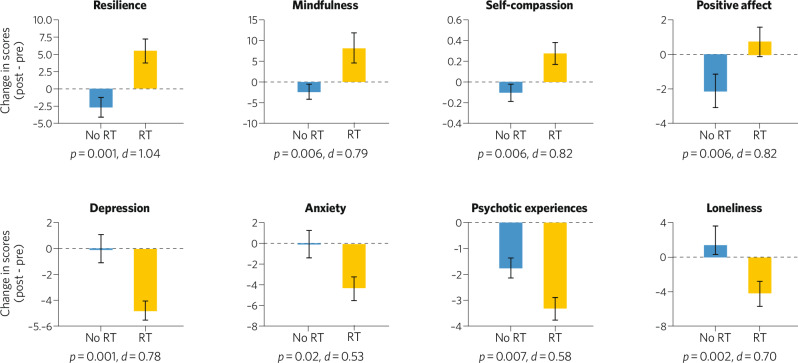
One example of a transdiagnostic preventive behavioral intervention that targets several resilience-associated capacities. Resilience Training (RT) consists of four 90-minute group sessions that teach young people mindfulness, self-compassion and mentalization skills. In a waitlist-controlled RCT, college students with either subclinical psychotic symptoms or symptoms of depression were randomized to either the active intervention (Resilience Training, RT, *n* = 54) or a waitlist control condition (No RT, *n* = 53). Significantly larger increases in resilience-related capacities, such as mindfulness, self-compassion, positive affect and overall resilience (measured using validated self-report scales) were observed in the RT compared to the waitlist condition. In addition, these improvements were associated with the significantly greater decreases in symptoms of psychopathology (depression, anxiety and psychotic experiences), and loneliness observed in the RT compared to the waitlist control condition. See [55, 56] for additional details.

There are a few indicated preventive interventions that have explicitly focused on measuring and augmenting protective factors, as well as symptom reductions. For example, Resilience Training (RT) is a four-session, mindfulness-based intervention that was designed for young adults experiencing depressive symptoms and/or psychotic experiences (subclinical psychotic-like symptoms) [103]. In a waitlist-controlled RCT conducted in 107 college students, significant increases in resilience-related capacities, including mindfulness, self-compassion and positive affect, as well as significant reductions in symptoms of psychopathology, such as psychotic experiences, depression, anxiety and loneliness, were observed in the young adults who received RT compared to those assigned to the waitlist control group [104, 105] (Fig. 1). Transdiagnostic interventions such as RT that focus on augmenting mental health-promoting capacities, behaviors or intrinsic strengths [58] may have more durable effects, as well as potentially greater appeal to non-help-seeking youth, than interventions that focus solely on reducing symptom-related biases and impairments.

### Indicated prevention for youth at risk for serious mental illnesses

In parallel to the large body of research focused on the prevention of internalizing disorders such as depression, there has been a substantial effort focused on developing and testing interventions that may prevent the onset of schizophrenia and related psychotic disorders, primarily in young people with subclinical psychotic symptoms [10]. These programs have most frequently used CBT, sometimes in combination with other treatments [10]. Overall, this body of research has produced mixed results, with limited evidence that these interventions lead to the prevention of psychotic disorders specifically [10, 106]. However, based on the evidence that subclinical psychotic symptoms are associated with elevated risk for the development of a number of psychiatric disorders and poor outcomes [107–109], in addition to psychosis, this research effort has now broadened to focus on a wider range of transdiagnostic risk states and outcomes [17, 110, 111]. For example, given that a substantial portion of individuals experiencing risk states defined by the presence of subclinical psychotic symptoms (i.e., the Clinical High Risk (CHR) or Ultra High Risk (UHR) for psychosis syndromes) have comorbid mood or anxiety disorders or develop these disorders over time, and experience varying degrees of functional impairment [112], intervention studies conducted in these populations have begun to include transdiagnostic outcomes.

One recent study of youth meeting criteria for UHR for psychosis which broadened the targeted outcomes beyond psychosis onset is the Staged Treatment in Early Psychosis (STEP) study. This study enrolled 342 UHR for psychosis youth (12-25 years old) in a sequential, multiple assignment randomized trial [111]. In this trial, the UHR participants were randomized to one of two interventions at three stages, advancing to the next stage if there was no treatment response at the prior stage (the interventions included: support and problem solving, CBT-based case management, fish oil, and an antidepressant or antipsychotic medication). The primary (transdiagnostic) outcome was global functioning. UHR remission rates and transition to psychosis were also measured. Possibly due to the high drop-out, low adherence and low fidelity rates in this trial, there were no significant differences between the treatment conditions in effects on the outcomes, for all three intervention stages. The low remission rates in this study are consistent with prior data showing that UHR individuals are often chronically quite symptomatic [112], and thus may not represent ideal targets of primary prevention approaches [65]. However, this trial can provide one overall model for staged-based care of high risk youth that could be employed to test interventions that might have specific effects on candidate psychological or neurobiological mechanisms that are variably affected within heterogeneous at-risk samples.

### The clinical staging approach

A related model of care that involves focusing on both: 1) a transdiagnostic at-risk population and 2) transdiagnostic outcomes, is the clinical staging model developed in Australia by McGorry, Hartmann and colleagues. This approach focuses on a broadly defined at-risk syndrome, referred to as the Clinical High At-Risk Mental State (CHARMS), that encompasses clinical risk phenotypes for four disorders: depression, bipolar disorder, borderline personality disorder, and psychosis [110, 113]. This model defines different stages of risk and severity, with Stage 0 including individuals with some form of risk who are without any distress-causing psychopathology, while Stages 1a and 1b require the presence of subsyndromal, distress-causing symptoms (with 1b corresponding to greater syndromal specificity than 1a and includes the CHARMS category). Stages 2-4 indicate the presence of a suprathreshold psychiatric disorder, with varying levels of severity and chronicity. An assumption of this model is that increasing levels of diagnostic specificity (clinical differentiation) emerge as clinical progression occurs across the stages in a subset of those at risk.

One longitudinal study of this staging model found that, of 1,370 youth at Stage 1b, 176 (12.8%) transitioned to a Stage 2 (full-threshold) disorder over a median follow up period of 14 months [114]. Another, smaller study that followed 41 Stage 1a and 80 Stage 1b youth over one year found that 28% of the Stage 1b youth developed a Stage 2 disorder (with 96% developing major depressive disorder), whereas only 9% of the Stage 1a youth transitioned to Stage 2 [110]. These initial findings suggest that the broader inclusion and outcome criteria of this model may be useful (i.e., associated with sufficient power) for testing transdiagnostic prevention approaches. However, potential heterogeneity in the psychological and neurobiological mechanisms associated with the early stages could lead to challenges in identifying specific interventions that consistently modify transition rates. Identifying Stage 1a or 1b individuals that show evidence for altered psychological processes that are shared across different forms of psychopathology [18], and testing interventions that target such processes in these individuals, could represent a rational next step for validating this transdiagnostic staging model.

## Combined approaches

Another way to increase statistical power and the potential to observe beneficial effects of a preventive intervention is to enrich the target sample for severity or types of risk. Some transdiagnostic prevention programs have employed this strategy by using several eligibility criteria, so that they essentially represent a combination of selective and indicated prevention strategies. This approach of targeting youth with multiple risk factors for developing psychiatric disorders may prove to be particularly cost effective, given the highly elevated level of transdiagnostic risk observed in these vulnerable populations.

For example, DBT [115] has been studied as a preventive intervention for children with a history of maltreatment (selective prevention); these children often present with a range of symptoms, including suicidal thoughts and behaviors (indicated prevention). DBT has shown effectiveness in preventing depressive symptoms, suicidal thoughts and behaviors, and improving emotion regulation skills in this high risk population [116–118].

This type of risk enrichment approach was also used in a study of a preventive intervention currently called Living In Families with Emotions (LIFE), which was developed for adolescents (age 11-14 years) with environmental risk factors (selective prevention), plus subclinical symptoms of psychopathology (indicated prevention). LIFE is a group-based intervention, consisting of eight sessions for the at-risk adolescents and three sessions for the adolescents’ caregivers, which was initially designed for adolescents living in an immigrant community with high levels of poverty and family disruption. The sessions focus on teaching emotion awareness and regulation techniques, as well as social skills. In a one arm pilot trial, enrolled children scored in the subclinical range on a mental health screening survey administered during the child’s annual check-up with their pediatrician. The study found that significant improvements in subclinical symptoms of anxiety and depression, social functioning, and emotion recognition occurred following the intervention [119].

## Digital approaches

Recent advances in digital mental health technologies have dramatically impacted the development and implementation of mental health interventions and may be useful for the early detection and prevention of psychiatric illnesses as well, given the importance, in prevention efforts, of minimizing burden and maximizing access. The development and use of smart phone-based applications, ecological momentary interventions, virtual reality (VR)-based programs, and artificial intelligence-enhanced screening approaches are quickly leading to substantial changes in how mental health interventions are conceptualized and delivered [120–124]. These changes include both adaptations of existing in-person interventions for digital delivery and the development of novel digital interventions, as well as hybrid (with digital and non-digital components) programs. Advantages of these programs include their convenience, the ability to tailor them to an individual’s specific needs, and the anonymity that can be provided in some cases. (Below we have described some interventions that have been tested primarily in adults or clinical populations, since they may represent novel approaches that could be applied in at-risk youth in the future, in addition to describing programs designed for youth.)

One digital intervention that was designed to reduce symptoms of anxiety and depression (indicated prevention) is an application that can be used on a range of devices (e.g., smartphone, computer, iPad) called ICare Prevent [125]. This program is comprised of 7 sessions (focused on problem solving, exposure, behavioral activation, psychoeducation, and cognitive restructuring among other topics), plus an additional booster session one month after completion of the 7 sessions. There are also 8 optional sessions focused on topics such as relaxation, substance use, and sleep. An RCT of ICare Prevent with 566 adults experiencing subclinical depression and/or anxiety were randomized to one of three conditions: 1) receiving the intervention with individual guidance (messaging) from a live coach; 2) receiving the intervention with automated guidance (standardized feedback following each session and no individual coaching) or 3) a waitlist. Significant reductions in anxiety and depression were observed in both treatment conditions, compared to the waitlist condition, up to six months later, with no significant differences between the live and automated guidance conditions [126].

An example of a hybrid (digital and non-digital delivery) indicated preventive intervention is the EMIcompass program, which uses an ecological momentary intervention (EMI) with an interactive approach. This program includes both face-to-face sessions and compassion-focused EMI-delivered tasks that participants complete each week using their mobile phone application. In one RCT of this program, EMIcompass was compared to treatment as usual (TAU) in 92 participants (ages 14-25) who were at-risk (with subclinical symptoms) or in the first-episode stage of a psychotic illness. While EMIcompass did not lead to significant improvements in the primary outcome, self-reported distress, stress reactivity was significantly reduced, and quality of life was improved, in the active compared to the TAU condition [127].

It is also important to note that there are several popular commercially available smartphone-based mental health-focused applications, such as Headspace and Calm, that have millions of monthly users [128]; while these applications do not have a specific prevention focus, they are universally available and widely used—thus they could serve as a universal prevention tool. While Calm has yet to be tested via RCTs, several RCTs have been conducted to assess the efficacy of Headspace in different populations, with most showing medium effect sizes in reducing symptoms of depression and anxiety [129].

## General considerations and future directions

### Comparison of strategies

Universal, selective, and indicated approaches for transdiagnostic prevention of mental illnesses have different strengths and limitations. The potential scalability of universal prevention programs is a key strength of this approach, particularly in light of the ongoing shortage of mental health care providers for youth [130, 131]. Regarding selective prevention, existing evidence suggests that children with histories of early adversity or with parents (or other relatives) with psychiatric disorders are populations that can benefit from prevention-focused interventions delivered early on, when mild stressors or very low levels of subclinical symptoms, or no symptoms, are present. Indicated interventions show the strongest evidence for efficacy, but they may not be feasible in some settings, since mental health screenings (a necessary first step for most indicated prevention programs) can be costly and labor intensive, and stigma or privacy-related concerns can limit their use.

However, in settings where mental health screening has become commonplace and accepted, e.g., in many pediatric/primary care clinics and some schools, indicated preventive interventions could be provided to mildly symptomatic youth if the necessary resources are available, potentially focused on prevention of transdiagnostic outcomes (e.g., need for individual mental health care, behavioral issues, school functioning). Indicated prevention programs may be associated with a higher likelihood, compared to universal and selective prevention approaches, that a substantial portion of the targeted population will benefit from the intervention, since shared psychological mechanisms (e.g., disruptions in emotion regulation, maladaptive cognitive biases) that are affected across individuals with different manifestations of subsyndromal psychopathology can be targeted by these interventions. Although the relative value of these three different strategies has been extensively debated, there is some emerging consensus that a combination of these approaches may provide the most benefit to youth (and society) as a whole [65].

### Transdiagnostic functional outcomes

It is somewhat surprising that there have been few studies to date that have measured the effects of preventive interventions on transdiagnostic *functional* outcomes, such as academic performance, school retention/drop out rates, social functioning, or transitions to college/employment, in addition to measuring symptoms of psychopathology or the onset of a psychiatric illness. Study designs that focus on functioning could capitalize on a shared feature of most categorically-defined psychiatric diagnoses – that they are often distinguished from subclinical states by their detrimental effects on functioning. Thus, maintenance of normative school and social behaviors, and achieving key developmental milestones, could serve as the long-term, “real world” goals of transdiagnostic intervention programs.

**Fig. 2:**
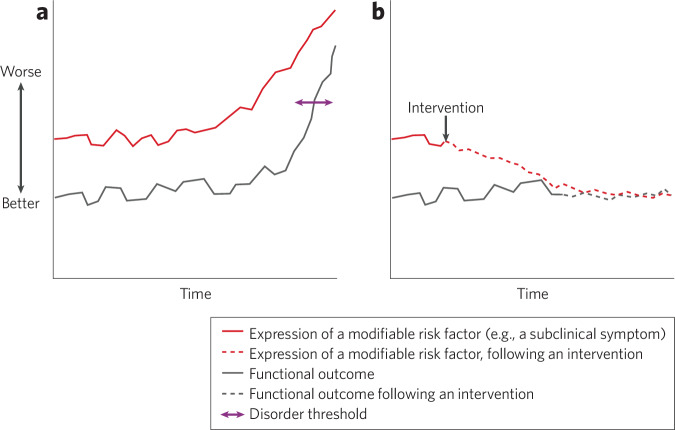
Schematic illustration of the relationship between a modifiable risk factor and an adverse long-term outcome, and effects of a preventive intervention on these targets. This illustration displays predicted changes in the expression of a modifiable risk factor, such as a subclinical symptom, and the longer-term effects of this risk factor, leading to a negative (undesirable) outcome, such as an impairment in functioning or the onset of a psychiatric disorder. In the absence of an intervention (panel **a**), the expression of the risk factor may increase over time (solid red line) and the negative outcome (functional impairment in this illustration) may emerge at a certain point (solid black line). Above a specific level of functional impairment (purple line), a disorder may be diagnosed. However, following a successful preventive intervention (panel **b**), the expression of the modifiable risk factor is reduced (dashed red line), and subsequently, at a later point in time, the negative outcome (e.g., functional impairment) is avoided (dashed black line).

Longitudinal studies are needed to assess transdiagnostic endpoints; measuring whether infrequently occuring outcomes, such as the onset of a serious mental illness or a precipitous decline in functioning, emerge over time requires monitoring over an extended period of development. In such studies, understanding the mechanistic relationships between the effects of the intervention on a *modifiable risk factor*, such as a subclinical symptom of psychopathology or a neurobiological mechanism, and the intervention’s longer-term preventive effects on the *targeted clinical or functional outcome*, such as the absence of onset of a certain diagnosis, category of diagnoses or functional decline related to a psychiatric condition (see Fig. 2 for an illustration of these relationships) will be necessary for developing targeted, individualized intervention strategies.

### Targeting individual-level mechanisms

Future studies may increasingly employ novel, individualized approaches that identify those who are most likely to respond to a given preventive intervention, and provide the skills or strategies that are most likely to be effective at reducing those individuals’ level of risk [132]. As has been suggested previously, progress towards achieving this goal would be accelerated by the validation of biological markers of risk that could be used to determine eligibility for prevention trials, and subsequently used as objective targets of interventions designed to modify that target. Establishing such markers has been an elusive goal of psychiatric neuroscience research for decades. However, some progress has occurred via several lines of research.

First, evidence for a set of common genetic variants that are associated with risk for multiple psychiatric disorders, as well as evidence for changes in the brain that are shared across psychiatric disorders [18], suggest that, ultimately, a “transdiagnostic risk profile” could be generated for individuals. This risk profile could also incorporate transdiagnostic environmental risk factors (e.g., prenatal exposures to toxins or infections, history of early adversity) and psychological features (e.g., aspects of temperament, personality) to identify those with different levels and types of risk.

Second, studies have been conducted which directly measure the effects of interventions on brain functioning, and some of these effects on the brain appear to correlate with symptom improvements associated with the intervention. These intervention-linked changes in brain functioning could be used to identify individuals who are most likely to benefit from an intervention. For example, meta-analyses have found evidence that a range of different types of psychotherapy, including mindfulness-based interventions [133], influence the function and connectivity of the “salience network” of the brain, which includes the anterior insula and dorsal anterior cingulate cortex, particularly in people with internalizing disorders [134, 135]. Effects of psychotherapy on the insula have also been observed in individuals with subthreshold symptoms of depression [136]. In addition, there is evidence that abnormal salience network activity at baseline may predict responses to psychotherapy [137]. These findings are particularly notable in light of evidence that the salience network may represent a common (transdiagnostic) site of changes in brain structure and function observed across multiple psychiatric disorders [18, 138–140]. Thus, future prevention trials could target individuals with non-specific risk factors for mental illness who also show changes in the salience network, to test whether a preventive behavioral intervention could modify these changes and risk for psychopathology. This strategy is broadly consistent with the mechanism-focused, “experimental therapeutics” approach to clinical trials that has been advocated by the National Institute of Mental Health [141].

Mindfulness intervention-related changes have also been observed in the functional connectivity between: 1) the default mode network, which is involved in self-awareness and introspection, and 2) brain networks that support cognitive control and interoceptive awareness (the central executive control and salience networks) [142, 143]. These circuits are involved in emotional regulation, among other functions [144–147], which may account for the changes observed in these networks following acquisition of mindfulness-related skills. An example of this type of finding is preliminary evidence for a link between 1) a reduction in emotion reactivity and 2) an increase in the functional connectivity between the hippocampus (a component of the default network) and the dorsolateral prefrontal cortex (a region involved in emotion regulation and cognitive control) in at-risk young adults following a mindfulness-based intervention [148].

In summary, these findings generally suggest that neuroimaging-based (or related behavioral) markers should be tested further for their utility in identifying individuals *within* at-risk populations, that are initially characterized by non-specific risk factors (e.g., a family history of mental illness, childhood adversity, mild subsyndromal symptoms), who could most benefit from preventive interventions that engage the relevant neural circuits.

### Depression as a broadly transdiagnostic target

Another potential next step in the overall effort to identify effective transdiagnostic prevention strategies is to re-purpose what is already known about treating and preventing certain common forms of psychopathology, using a transdiagnostic lens. For example, meta-analyses have established that major depressive disorder can be prevented in a portion of at-risk individuals using psychological [149–151] and exercise [152, 153] interventions. Also, numerous lines of research have shown that, across different individuals, depression can represent either an *endpoint* (the final stage of the evolution of a mental illness for an at-risk individual) or an *intermediate stage* along a trajectory towards the development of another chronic mental illness such as bipolar disorder or schizophrenia [154–158]. Similarly, anxiety in children and adolescents has been linked to an increased risk for the later development of a range of psychiatric disorders [159, 160], thus could also serve as a marker of an intermediate stage of transdiagnostic risk for mental illnesses for some individuals. This is consistent with the fact that certain cognitive or affective traits associated with depression and anxiety, such as negative attribution biases, are common in the general population and confer risk for multiple psychiatric disorders. Thus, targeting depression, internalizing symptoms (depression and anxiety), or cognitive styles associated with these symptoms, could represent a transdiagnostic prevention strategy that may be effective in reducing risk for multiple psychiatric conditions.

### Systemic and developmental considerations

Overall, a great need remains for evidence-based transdiagnostic preventive interventions that are proven to be scalable, minimally burdensome, and can engage young people “where they are” both literally (i.e., in spaces where youth spend their time) and culturally (using youth-friendly language, images, activities and technology). Several pioneering examples of successful community-based youth mental health programs [161–163] have begun to demonstrate the feasibility of these types of approaches. In addition, some primary care programs have expanded to provide mental health services, and, while most of these programs do not deliver a specific mental health-focused intervention, they tend to serve a wide range of youth and provide mental health assessments along with medical care [164]. For example, the “headspace” program, which has more than 65 sites in Australia, combines primary care with mental health-related (indicated) preventive interventions for youth who may be at risk for a mental illness [165], offering multiple avenues of care, ranging from phone hotlines that provide access to a clinician, to support for those seeking employment, individual mental health treatment, and family services.

To maximize retention of youth in prevention-focused programs, consideration of the developmental stage (e.g., the education level and psychological capacities) of the youth is important when designing the content and structure of interventions, including decisions about the length of sessions, number of breaks, degree of parent/caregiver involvement, and the overall strategy for recruitment. For example, there is some evidence that the effectiveness of preventive interventions in youth is enhanced by parental involvement [166–168]. On the other hand, older adolescents may be reluctant to participate in a program that involves their parents [169]. For young adults, typically their engagement and some degree of immediate benefit from an intervention must be substantial in order to maintain their participation, since their time and attention is often consumed by a variety of social, academic and online activities and, unlike children and adolescents, they have the autonomy to choose (or decline) to participate in a mental health program. Also, given that subclinical psychopathology may become more differentiated (less transdiagnostic) in some at-risk youth over time, the need for indicated interventions may become greater during late adolescence/early adulthood, whereas universal interventions may be most impactful for pre-adolescent children [170]. However, these and other developmental considerations require further study.

### Shifting public opinion

Further education of the public and policy makers regarding the value of screening and tiered prevention programs for enhancing youth mental health is greatly needed to accelerate progress. Highlighting similarities to successful prevention programs employed in other fields of medicine may provide an initial rationale for investing resources in avoiding, interrupting, or slowing the progression of mental illnesses in vulnerable young people. As increasingly rigorous and well-powered trials are conducted, a strong empirical justification and related motivation for disseminating routine preventive mental health care for youth may grow over time.
